# Transcriptome Analysis and Identification of Insecticide Tolerance-Related Genes after Exposure to Insecticide in *Sitobion avenae*

**DOI:** 10.3390/genes10120951

**Published:** 2019-11-20

**Authors:** Ning Wei, Yongzhi Zhong, Lulu Lin, Minghui Xie, Guangling Zhang, Weihua Su, Chuanren Li, Haoliang Chen

**Affiliations:** 1 College of Agriculture, Yangtze University, Jingzhou 434025, Hubei Province, China; 201671408@yangtzeu.edu.cn; 2 Institute of Plant Protection and Agro-Products Safety, Anhui Academy of Agricultural Sciences, Hefei 230031, Anhui Province, China; zhongyongzhi@aaas.org.cn (Y.Z.); linlulu@aaas.org.cn (L.L.); xieminghui@aaas.org.cn (M.X.); zhangguangling@aaas.org.cn (G.Z.); suweihua@aaas.org.cn (W.S.)

**Keywords:** *Sitobion avenae*, RNA-seq, differentially expressed unigenes, insecticide tolerance-related genes

## Abstract

Aphids cause serious losses to the production of wheat. The grain aphid, *Sitobion avenae,* which is the dominant species of aphid in all wheat regions of China, is resistant to a variety of insecticides, including imidacloprid and chlorpyrifos. However, the resistance and mechanism of insecticide tolerance of *S. avenae* are still unclear. Therefore, this study employed transcriptome analysis to compare the expression patterns of stress response genes under imidacloprid and chlorpyrifos treatment for 15 min, 3 h, and 36 h of exposure. *S. avenae* adult transcriptome was assembled and characterized first, after which samples treated with insecticides for different lengths of time were compared with control samples, which revealed 60–2267 differentially expressed unigenes (DEUs). Among these DEUs, 31–790 unigenes were classified into 66–786 categories of gene ontology (GO) functional groups, and 24–760 DEUs could be mapped into 54–268 Kyoto Encyclopedia of Genes and Genomes (KEGG) pathways. Finally, 11 insecticide-tolerance-related unigenes were chosen to confirm the relative expression by quantitative real-time polymerase chain reaction (qRT-PCR) in each treatment. Most of the results between qRT-PCR and RNA sequencing (RNA-Seq) are well-established. The results presented herein will facilitate molecular research investigating insecticide resistance in *S. avenae*, as well as in other wheat aphids.

## 1. Introduction

Wheat, *Triticum aestivum*, is considered one of the most important cereals in China, as well as around the world. Aphids cause losses of more than 10% of the harvest in an average year and over 30% in years with serious damage [1]. The grain aphid, *Sitobion avenae*, is the dominant aphid in all wheat regions of China and is also responsible for the greatest loss to wheat production [2]. Both the aphid adult and nymph feed on the wheat leaves, stems, and ears, causing the plants to stunt, be unable to ear, or even die. Aphids also damage wheat by the transmission of plant viruses, such as barley yellow dwarf virus, and impact photosynthesis by the production of honeydew, all of which lead to wheat yield loses and poor quality [1,2,3].

In China, insecticides treatment is the most prevalent management strategy used against grain aphids when planting wheat. Identifying the specific genes involved in insecticide detoxification and their genetic pathways could be beneficial for controlling *S. avenae*. However, studies of the insecticide detoxification of grain aphids arelimited. RNA-Seq is now a common method used to analyze gene expression [4], and this method has been used to identify insecticide metabolism-related genes in several species, including *Aedes aegypti* [5], *Bradysia odoriphaga* [6], and *Plutella xylostella* [7]. There are no transcriptome analyses about *S. avenae* under insecticide pressure that have been conducted to date [8,9]. Therefore, clarifying the genes involved in insecticide detoxification will be beneficial for controlling this particular pest if it develops resistance to a single or group of insecticides.

In this study, we attempted to identify the specific genes’ response to insecticides of *S. avenae* using next-generation sequencing technologies. We generated the annotated transcriptome of the grain aphid first and then used RNA-Seq to analyze the gene responses to insecticides. The data generated in this study provide abundant resources based on directed sequencing that will be useful to our understanding of the molecular insecticide tolerance of *S. avenae* and provide us with new thoughts for the pest management strategy.

## 2. Materials and Methods

### 2.1. Insect and Chemicals

Grain aphids were collected from the wheat field experimental practice base of the Anhui Academy of Agricultural Sciences (117.21 °E, 31.96 °N), then reared on wheat seedlings to establish an aphid colony in the laboratory. Briefly, 40−60 wheat seeds were steeped in water for 24 h, then planted in 10 cm diameter plastic bowls with nutrient soil. Aphids were introduced to wheat seedlings when they emerged for 4 days. Bowls of seedling with aphids were placed in a mesh cage (length: 30 cm; width: 30 cm; and height: 30 cm). Every week during culture, the leaves containing the aphids were removed and placed into bowls with fresh seedlings. Grain aphids were reared in a walk-in chamber at 23.0 °C under a 16:8 L: D photocycle and 50–70% relative humidity.

The insecticides used in this study were technical grade imidacloprid (97% purity, Hubei Sanonda Co., Ltd., Jingzhou, Hubei, China) and chlorpyrifos (96% purity, Jiangsu Lanfeng Biochemical Co., Ltd., Xuzhou, Jiangsu, China). Acetone (analytically pure, Suzhou Chemical Reagent Co., Ltd., Suzhou, Jiangsu, China) was used as a solvent to dissolve the pesticides to the application concentrations.

### 2.2. Bioassay

Bioassays were conducted as described by Lu et al. [10] to estimate grain aphid LC_50_. Briefly, imidacloprid and chlorpyrifos were dissolved in acetone as a stock solution at 2000 mg/L and 1000 mg/L, respectively. The stock solution was then diluted to 5−7 different concentrations, after which 0.2 mL aliquots of each dilution were placed in clear glass vials with an inner area of 47.1 cm^2^ (15 mm in diameter and 100 mm in length). The vials were immediately rolled for 5 min to uniformly distribute the residue on the inner surface, after which they were motionless for 1 h to allow acetone to evaporate and then capped with absorbent cotton. The vials with the insecticide films were subsequently used for toxicity tests, and vials treated with acetone were used as controls. To test the toxicity, 20 healthy apterous aphid adults of similar shape were introduced into each vial, and each treatment had three replicates. Tests were conducted in a walk-in chamber at 23.0 °C under a 16:8 L:D photoperiod and 50–70% relative humidity. Mortality was determined 3 h later, and grain aphids were considered dead when no leg or only one leg moved when touched with a soft brush. Insecticide-treated mortality was considered to have occurred when the mortality of the control was <10%. Bioassay data were analyzed by using Probit in SPSS 16.0 software [11].

### 2.3. Sample Preparation

Chlorpyrifos and imidacloprid stock solution diluted with acetone to an LC_10_ concentration according to the bioassay result, after which 0.2 mL aliquots of the diluted pesticide solutions were coated onto clear glass vials as described above. Next, 20 healthy apterous aphid adults of similar shape were placed into vials coated with the insecticide residue for 15 min, 3 h, and 36 h. To ensure there were enough live grain aphids after treatment, there were six replicates in each group. Fifteen aphids that survived in each treatment were collected as one sample for RNA-Seq. Grain aphid apterous aphid adults treated with acetone for 15 min were collected as controls. Each treatment for RNA-Seq was conducted in triplicate. In total, 21 samples were collected for RNA-Seq.

### 2.4. RNA Isolation, cDNA Library Construction, and Illumina Sequencing

Total RNA isolation for insecticide-treated and control aphids was conducted using oligo dT attached magnetic beads. Genomic DNA was removed using DNAse I (TaKaRa, Dalian, China). The integrity and purity of total RNA were determined using a 2100 Bioanalyzer (Agilent Technologies, Santa Clara, CA, USA) and quantified with an ND 2000 (NanoDrop Thermo Scientific, Wilmington, DE, USA). The RNA integrated values of the samples chosen for RNA-Seq were between 6.4 and 6.7, while the OD260/280 value of those samples was between 1.93 and 2.09. The mRNA was randomly interrupted to short fragments by adding fragment buffer, and these short fragments were utilized as templates for synthesis of double-strand cDNA using a SuperScript double-strand cDNA synthesis kit with random hexamer primers (Illumina, San Diego, CA, USA). The synthesized cDNA was subjected to end repair, phosphorylation, and “A” base addition, according to Illumina’s library construction protocol. Libraries were size selected for cDNA target fragments of 200–300 bp on 2% Low Range Ultra Agarose, then PCR amplified using Phusion DNA polymerase (New England Biolabs, Boston, MA, USA) for 15 PCR cycles. After quantification by TBS380, RNA-Seq libraries were sequenced on an Illumina Hiseq X ten sequencer (Illumina, San Diego, CA, USA) for 2×150 bp paired-end reads.

### 2.5. Sequencing Data Analysis

SeqPrep (https://github.com/jstjohn/SeqPrep) and Sickle (https://github.com/najoshi/sickle) with default parameters were used to trim and control the quality of raw paired reads. The clean data of insecticide-treated and control samples were used to conduct de novo assembly with Trinity (http://trinityrnaseq.sourceforge.net/) [12]. The assembled transcripts were searched against the NCBI protein nonredundant (NR), Swiss Prot, Pfam, and Cluster of orthologous groups (COG) databases using BLASTX, the proteins that had the highest sequence similarity with the given transcripts were used to retrieve their function annotations, and typical cut off E values less than 1.0×10^−5^ were set. Gene ontology (GO) annotations of unique assembled transcripts for describing biological processes, molecular functions, and cellular components were generated using BLAST2GO (https://www.blast2go.com/) [13]. The Kyoto Encyclopedia of Genes and Genomes (KEGG, http://www.genome.jp/kegg/) [14] was used to perform metabolic pathway analysis. The raw reads were deposited in the NCBI Sequence Read Archive database under accession numbers SRR8953735–8953755.

### 2.6. Analysis of Differential Expressed Unigenes (DEUs), GO Annotation, and Pathway Enrichment

Gene expressed levels were assessed by RSEM (RNA-Seq by Expectation Maximization) [15] for each sample using the basic method of clean data mapping back to the assembled transcriptome to obtain the read count of each gene. The DESeq2 was used to perform the DEUs’ analysis of control groups and insecticide treatment groups [16]. The relative expression of unigenes was calculated by dividing the unigene’s fragments per kilobase per million mapped reads (FPKM) value into insecticide-treated groups with the same unigene FPKM value in control groups. The R statistical package software EdgeR (Empirical analysis of Digital Gene Expression in R, http://www.bioconductor.org/packages/2.12/bioc/html/edgeR.html) was utilized for differential expressed analysis, and the R code for detect differential expression of unigenes between treated with imidacloprid for 15 min and control was provided in the Supplementary File S1 [17]. In addition, functional enrichment analysis, including GO and KEGG, was performed to identify which DEGs were significantly enriched in GO terms and metabolic pathways at Bonferroni corrected *p*-values < 0.05 when compared with the total transcriptome background. GO functional enrichment and KEGG pathway analysis were conducted using Goatools (https://github.com/tanghaibao/Goatools) and KOBAS (http://kobas.cbi.pku.edu.cn/) [18].

### 2.7. Sequence Confirmation and qRT-PCR Validation

Eleven DEUs assembled sequences, including one ABC transporter, one glutathione s-transferases (GST), one esterase, three cytochrome P450, two uridine 5′-diphospho-glucuronosyltransferase (UGT), three trypsin, and one reference gene nicotinamide adenine dinucleotide (NADH) were confirmed and the irrelative expressions validated by qRT-PCR. Primer premier 5.0 was used to design specific primers to confirm the assembled sequences, and then reverse transcription PCR was conducted (Supplementary File S2). PCR products were then analyzed by gel electrophoresis, and expected DNA bands were extracted using an Agarose Gel Extraction Kit (Takara Biotechnology (Dalian) Co., Ltd., Dalian, Liaoning, China). Then DNA was sub-cloned into the vector (pEASY-T1 Simple Cloning Kit, Beijing TransGen Biotech Co., Ltd., Beijing, China) according to the manufacturer’s protocols and a 3730 DNA analyzer was used to determine the nucleotide sequences. The confirmed sequences were deposited in the GenBank database under accession numbers MN481369 to MN481380.The relative expression of 11 confirmed sequences was validated by qRT-PCR, while NADH was chosen as the reference gene [19]. Beacon Designer 7.0 was used to design the specific primers for qRT-PCR (Supplementary File S2). One microgram total RNA was used to synthesize the cDNA after removal of genomic DNA (PrimeScript™ RT Reagent Kit with gDNA Eraser, Takara Biotechnology (Dalian) Co., Ltd., Dalian, Liaoning, China). SYBR Premix (Takara Biotechnology (Dalian) Co., Ltd., Dalian, Liaoning, China) was used for qRT-PCR conduct on a CFX96 Real-Time PCR Detection System (BioRad Laboratories, Inc., Hercules, CA, USA). The thermal cycling conditions were 45 cycles of 95 °C for 30 s, 58 °C for 30 s, and 72 °C for 30 s. The qRT-PCR was done accordingly to MIQE (minimum information for Q-PCR experiment) [20]. The amplification efficiency of each pair of specific primers was calculated (Supplementary File S3) [21]. Three biological samples, with two technical replicates, were used to determine the relative expression of unigenes. The NCBI accession numbers of confirmed sequences and the primers designed for confirmation and qRT-PCR are listed in File S2.

## 3. Results

### 3.1. Bioassay, RNA Sequencing, Assembly, and Annotation

**Figure 1:**
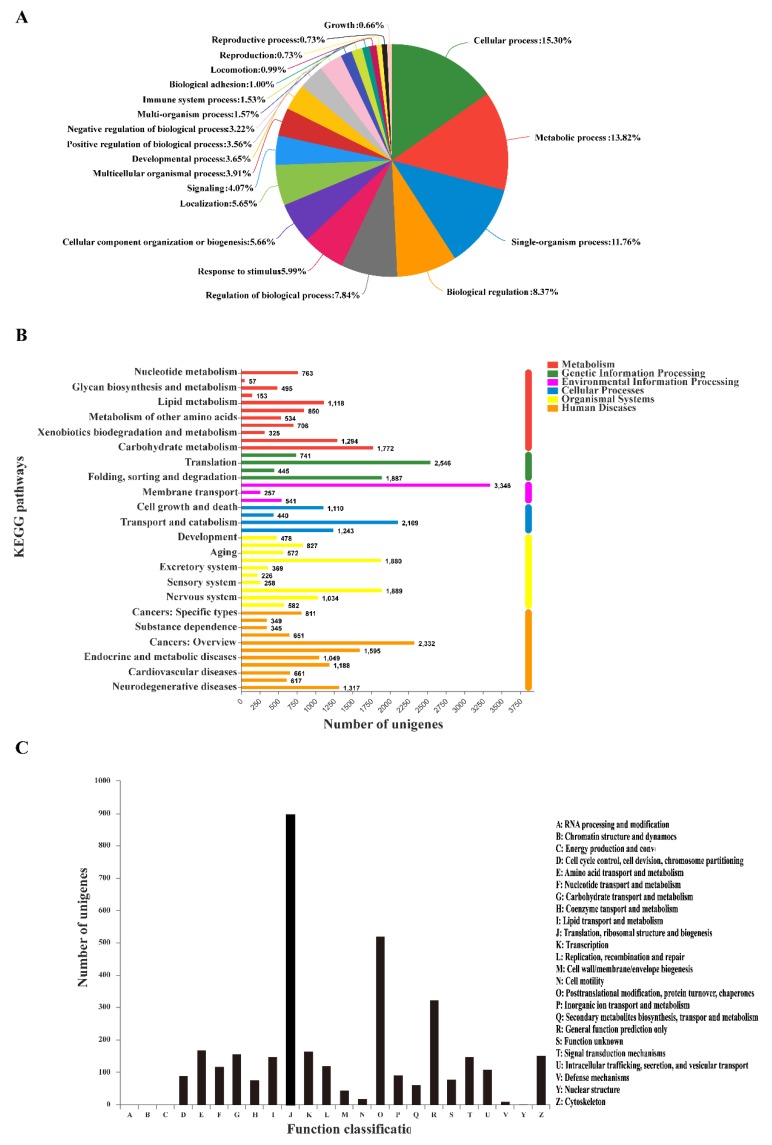
Results of unigenes against gene ontology (GO), Kyoto Encyclopedia of Genes and Genomes (KEGG), cluster of orthologous groups (COG) database. (**A**) Gene ontology annotation and classification of the *Sitobion avenae* transcriptome; (**B**) Kyoto Encyclopedia of Genes and Genomes annotation and pathways of the *S. avenae* transcriptome; (**C**) Cluster of orthologous groups function classification of the *S. avenae* transcriptome.

The bioassay results are shown in Table 1 and Supplementary File S4. The LC_50_ value of imidacloprid was about 109 times that of chlorpyrifos, indicating that chlorpyrifos is much more toxic than imidacloprid toward *S. avenae*. Overall, 21 samples, including insecticide-treated and control samples, generated 48,350,256 raw sequencing reads on average, of which 47,942,838 clean reads were obtained after filtering low-quality reads (Supplementary File S5). For all samples, the percentage clean reads ratio was higher than 99%, with an average of 96.81% meeting base call quality at Q20, indicating clean reads exhibiting good quality (File S5). The GC% ranged from 41.17% to 42.29%, with an average of 41.70% for all samples. All clean reads were then used to assemble an *S. avenae* unigenes reference database, which generated 134,474 unigenes. The unigenes database had a total length of 162,507,265 bp, a unigene length range of 201−27,421 bp, an average length of 874.44 bp, and an N50 of 1517 bp. The percentage of clean reads mapped to the assembly reference database ranged from 83.86% to 85.92%, with an average of 84.95% (File S5). The size distribution indicated that 23,970 unigenes were more than 1000 bp (Supplementary File S6). Annotation results showed that 46,690 (34.72%) of the 134,474 total unigenes can be annotated after against different databases. For different databases, we found 43,970 (32.70%), 32,505 (24.17%), 26,771 (19.91%), 7121 (5.29%), 22,338 (16.61%), and 26,887 (19.99%) unigenes were annotated in the NR, Swiss-Prot, Pfam, COG, GO, and KEGG databases, respectively. The top three functions among the GO annotations were cellular processes (15.30%), metabolic processes (13.82%), and single organism processes (11.76%) (Figure 1A). In the KEGG mapped results, the pathways were divided into six main categories, of which human diseases (10,915), organizational (8115), and metabolism (8067) accounted for the top three (Figure 1B, Supplementary File S7). In the COG, the largest number of annotations (903 unigenes) were translation, ribosomal structure, and biogenesis, which belonged to information storage and processing, followed by 521 unigenes annotated to posttranslational modification, protein turnover, and chaperones, which belonged to cellular process and signaling, and the third was 323 unigenes annotated to general function prediction only, which has been poorly characterized (Figure 1C, Supplementary File S8).

### 3.2. Unigene Expression Analysis for Insecticide Treatments

**Figure 2:**
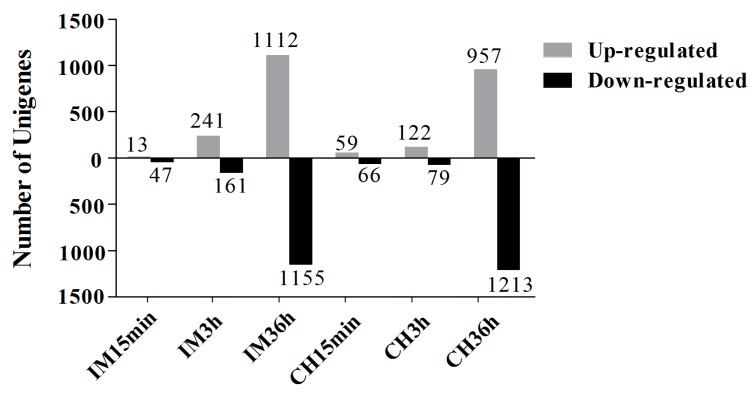
The number of up- and downregulated unigenes in samples of insecticide-treated compared with control. CK: control; CH: chlorpyrifos; IM: imidacloprid; 15 min, 3 h, and 36 h: treated with chlorpyrifos or imidacloprid for 15 min, 3 h, and 36 h, respectively.

**Figure 3:**
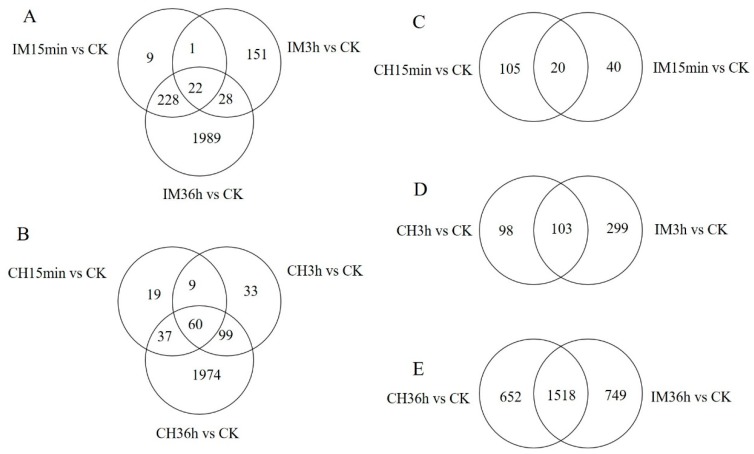
Venn map of the differentially expressed unigenes (DEUs) induced by treating adults with chlorpyrifos or imidacloprid for different durations. CK: control; CH: chlorpyrifos; IM: imidacloprid; and 15 min, 3 h, and 36 h: treated with chlorpyrifos or imidacloprid for 15 min, 3 h, and 36 h, respectively. (**A**) Intersection of DEUs in response to chlorpyrifos treatment for 15 min, 3 h, and 36 h compared with the control, (**B**) intersection of DEUs in response to imidacloprid treatment for 15 min, 3 h, and 36 h compared with control, (**C**) intersection of DEUs in response to imidacloprid and chlorpyrifos treatment for 15 min compared with the control, (**D**) intersection of DEUs in response to imidacloprid and chlorpyrifos treatment for 3 h compared with the control, and (**E**) intersection of DEUs in response to imidacloprid and chlorpyrifos treatment for 36 h compared with the control.

The identification of DEUs of insecticide-treated samples was compared with the control based on the FPKM value. Unigenes were considered DEUs only if the fold change (FC) expression ratios of insecticide-treated samples versus the control sample were larger than two or less than 0.5. A total of 60–2267 unigenes were considered DEUs after pesticide treatment (Figure 2). The number of DEUs increased as the insecticide treatment time increased. *S. avenae* treated with chlorpyrifos for 36 h had the most downregulated unigenes (1213), while the minimum number of downregulated unigenes (47) was observed in response to treatment with imidacloprid for 15 min. The maximum number of upregulated unigenes (1112) was observed in response to treatment with imidacloprid for 36 h, while treatment with imidacloprid for 15 min led to the minimum number of upregulated unigenes (13) (Figure 2, Supplementary File S9). The intersection of the DEUs Venn map for different insecticides treatment and different durations is shown in Figure 3. For the imidacloprid treatment, different time durations had 22 intersection DEUs (Figure 3A), while chlorpyrifos had 60 intersection DEUs (Figure 3B). However, for the same time duration, different insecticides treatments also had intersection DEUs. After 15 min, different insecticides treatment had 20 intersection DEUs (Figure 3C), while for 3 h, there were 103 intersection DEUs when treated with imidacloprid or chlorpyrifos (Figure 3D), and for 36 h, there were 1518 intersection DEUs when treated with imidacloprid or chlorpyrifos (Figure 3E).

### 3.3. GO Classification and KEGG Pathway Identification of DEUs

**Figure 4:**
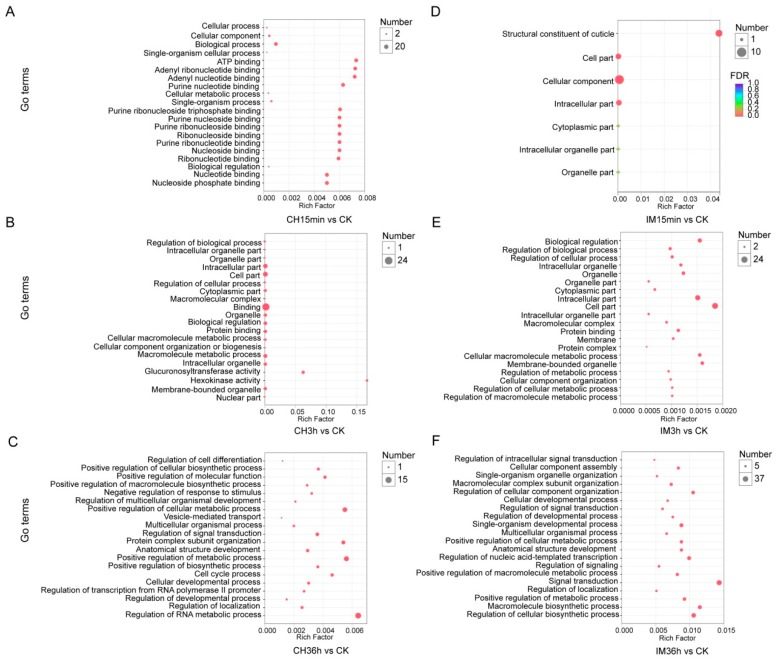
GO enrichment of DEUs induced by the treatment of adults with chlorpyrifos or imidacloprid for different durations. CK: control; CH: chlorpyrifos; IM: imidacloprid; and 15 min, 3 h, and 36 h: treated with chlorpyrifos or imidacloprid for 15 min, 3 h, and 36 h, respectively. (**A**) Treatment with chlorpyrifos for 15 min versus control, (**B**) treatment with chlorpyrifos for 3 h versus control, (**C**) treatment with chlorpyrifos for 36 h versus control, (**D**) treatment with imidacloprid for 15 min versus control, (**E**) treatment with imidacloprid for 3 h versus control, and (**F**) treatment with imidacloprid for 36 h versus control.

**Figure 5:**
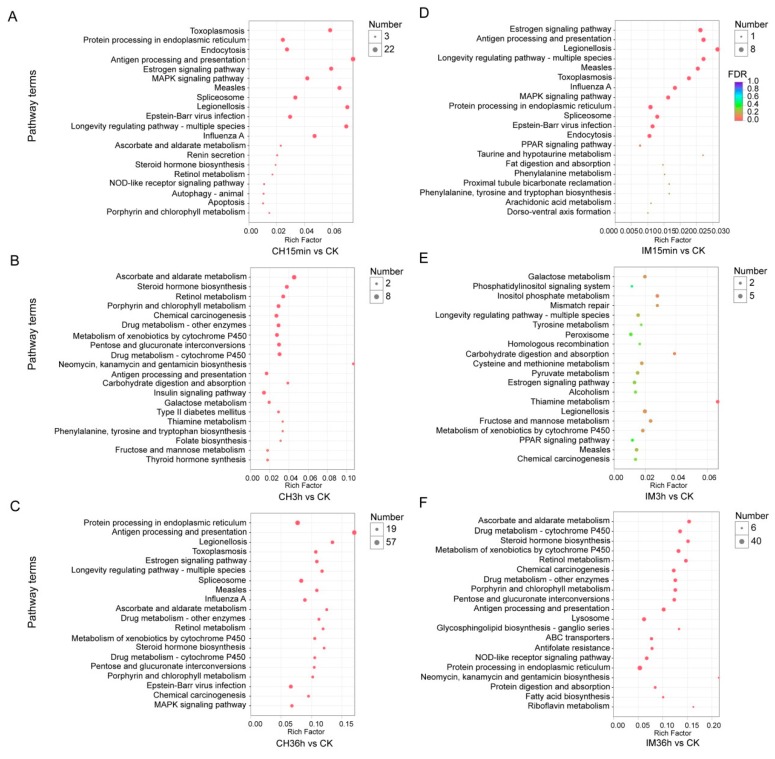
Pathway enrichment for DEUs induced by the treatment of adults with chlorpyrifos or imidacloprid for different durations. CK: control; CH: chlorpyrifos; IM: imidacloprid; and 15 min, 3 h, and 36 h: treated with chlorpyrifos or imidacloprid for 15 min, 3 h, and 36 h, respectively. (**A**) Treatment with chlorpyrifos for 15 min versus control, (**B**) treatment with chlorpyrifos for 3 h versus control, (**C**) treatment with chlorpyrifos for 36 h versus control, (**D**) treatment with imidacloprid for 15 min versus control, (**E**) treatment with imidacloprid for 3 h versus control, and (**F**) treatment with imidacloprid for 36 h versus control.

DEUs generated from different comparisons were assigned into GO term analysis. A total of 31–790 unigenes were classified into 66–786 categories of GO functional groups (Figure 4). The DEUs of imidacloprid treated for 36 h in comparison with the control had the largest number of unigenes that could be classified into GO terms, of which 790 unigenes were assigned into 786 GO terms. The DEUs of imidacloprid treated for 15 min had the lowest number of unigenes that could be assigned into GO terms, of which only 31 were categorized into 66 GO terms (Supplementary File S10). To identify possible active biological pathways of DEUs, the unigenes were mapped into the KEGG pathways. In the DEUs of different comparisons, 24−760 DEUs could be mapped into 54−268 KEGG pathways (Figure 5). The imidacloprid treatment for 36 h had the largest DEUs (760) mapped into the largest number of pathways (268), while imidacloprid treatment for 15 min led to the lowest number of DEUs (24) mapped into the least pathways (54) (Supplementary File S11). The ten most up- and downregulated DEUs after imidacloprid and chlorpyrifos treatment for 36 h compared with the control are listed in Table 2 and Table 3, respectively. A total of 18 out of 20 most significant DEUs have annotation result in the NCBI Nr database for imidacloprid treated compared with control, while 17 out of 20 most significant DEUs have annotation results for chlorpyrifos treated compared with control.

### 3.4. Insecticide Tolerance Related Unigenes Analysis and Quantitative Real-Time PCR (qRT-PCR) Validation

**Figure 6:**
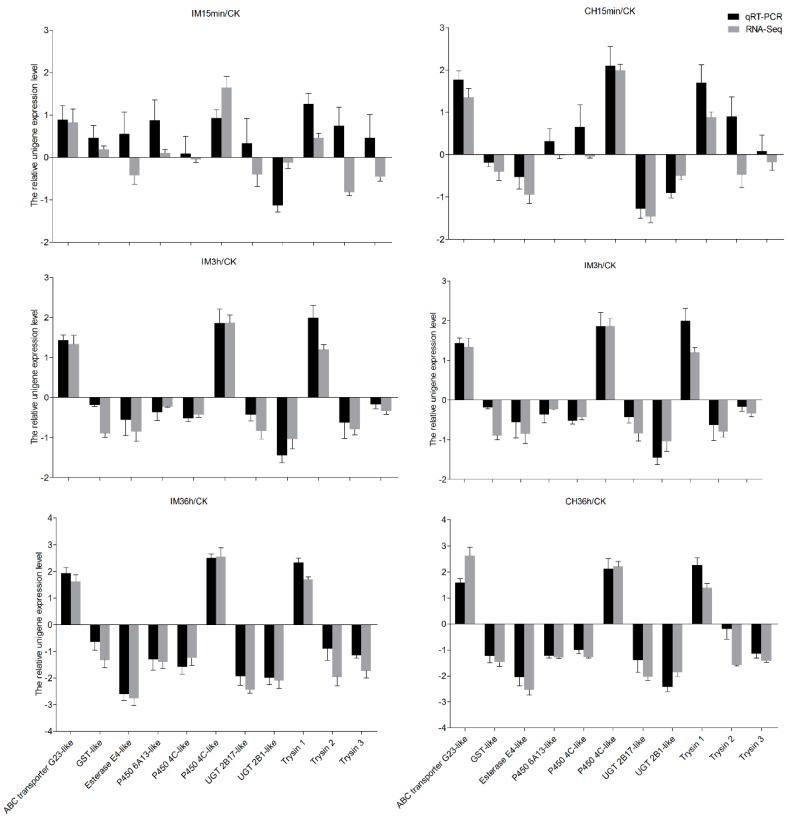
Comparison of the RNA-Seq and qRT-PCR results for the treatment of adults with chlorpyrifos or imidacloprid for different durations. CK: control; CH: chlorpyrifos; IM: imidacloprid; 15 min, 3 h, and 36 h: treated with chlorpyrifos or imidacloprid for 15 min, 3 h, and 36 h.

The DEUs of insecticide-treated and control samples that were considered to be insecticide tolerance-related unigenes are listed in Table 4. As the insecticide treatment time increased, the number of insecticide tolerance-related DEUs increased. After treatment with insecticides for 36 h, cuticle protein had the largest number of DEUs, followed by the ABC transporter and trypsin. After insecticide treatment for 36 h, the same 39 DEUs related to cuticle proteins were found in both the imidacloprid and chlorpyrifos treatment, while there were 50 DEUs in the imidacloprid treatment (four upregulated and 46 downregulated) and 43 DEUs in the chlorpyrifos treatment (three upregulated and 40 downregulated). ABC transporter possessed the second largest number of DEUs after insecticide treatment for 36 h (29 DEUs), with eight upregulated and 21 downregulated in the imidacloprid treatment and 25 DEUs with eight upregulated and 17 downregulated in the chlorpyrifos treatment. Among these DEUs, the same 19 DEUs related to ABC transporter were found in response to both the imidacloprid and chlorpyrifos treatment. Trypsin had the third-largest number of DEUs after insecticide treatment for 36 h, 32 DEUs with 15 upregulated and 17 downregulated were observed in response to imidacloprid treatment, while 20 DEUs with eight upregulated and 12 downregulated were observed in the chlorpyrifos treatment. Additionally, the same 20 DEUs related to ABC transporter were found in both the imidacloprid and chlorpyrifos treatment. For the metabolism enzyme-related unigenes, such as cytochrome P450, GST, and carboxylesterase, there were two, two, and 11 cytochrome P450 related DEUs observed in response to chlorpyrifos treatment for 15 min, 3 h, and 36 h, respectively, and the same two DEUs with one upregulated and one downregulated were found at all treatment times. There were two, seven, and 11 cytochrome P450 related DEUs observed in response to imidacloprid treatment for 15 min, 3 h, and 36 h, respectively, with one identical upregulated DEUs found in all time durations, and one identical downregulated DEU found in the 15 min and 36 h groups, as well as the same three upregulated DEUs found after 3 h and 36 h of treatment. DEUs related to GST were only observed after 36 h of treatment, and the same downregulated DEU was found in response to both imidacloprid and chlorpyrifos treatment. DEUs related to carboxylesterase were only observed after 36 h of treatment, and the same six downregulated DEUs were observed in response to imidacloprid and chlorpyrifos treatment (Supplementary File S12). Finally, the expressed levels of one ABC transporter, one GST, one esterase, three cytochrome P450, two UGT, three trypsin related DEUs were chosen for qRT-PCR validation. Nine of sixty-six comparisons of RNA-Seq and qRT-PCR results do not agree, but all of them appear in the treatment of 15 min (Figure 6).

## 4. Discussion

Insect resistance to pesticides is a complex impediment to agricultural production. Understanding how insects develop resistance to insecticides and the insecticide tolerance mechanism involved would help reduce and delay this process in insects. Using next-generation technologies to reveal tolerance and analyze insecticide-related genes by transcriptome profiles not only makes up for the gaps in previous studies but also provides us with new thoughts regarding insecticide resistance in *S. avenae*.

In this study, the number of DEUs increased as the insecticides’ treatment time increased, and the KEGG and GO pathways were more abundant. Furthermore, some insecticide-related genes were only differentially expressed after treatment with insecticides for 36 h, including glutathione s-transferase, carboxylesterase, acetylcholinesterase, acetylcholine receptor, chloride channel, and superoxide dismutase. Some genes may have been up- or downregulated when grain aphids were under insecticide pressure for 15 min and 3 h, but not significantly. After 36 h of insecticide treatment, they may have significantly up- or downregulated. In *B. odoriphaga*, when samples were treated with chlorpyrifos and clothianidin for 6 h and 48 h, the number of DEUs related to insecticide tolerance was not much different, and the majority of insecticide tolerance-related unigenes responded in 6 h; however, there were still some insecticide tolerance-related unigenes that responded in 48 h [6], which was similar to the results of the current study. Therefore, the results of this study may indicate that exposure to insecticides for 3 h does not lead to a great increase in tolerance-related unigenes. After 36 h of insecticide treatment, many more insecticide tolerance-related unigenes were differentially regulated, including those that responded to short-term exposure. These results indicated that the treatment duration of insecticides has a greater impact on DEUs related to insecticide tolerance than the type of insecticide.

Cytochrome P450 is an enzyme that has a variety of metabolic functions, and increased cytochrome P450 mediated drug metabolism is an important detoxification mechanism for insects [22]. The overexpression of the P450 monooxygenase enzyme is the most common mechanism of imidacloprid and chlorpyrifos resistance [23,24,25]. In aphids, P450 monooxygenase enzyme also plays an important role in insecticide detoxification and resistance [26,27,28]. In the present study, samples treated with chlorpyrifos showed five upregulated P450 unigenes, as well as a fold change in imidacloprid-treated samples. Four of the five unigenes were annotated as the CYP4Csubfamily gene, and one was annotated as the CYP6A subfamily gene. Unigene DN67665_c1_g4 showed upregulation with time, and unigene DN68255_c7_g3 showed the highest upregulated fold change (3.29). Furthermore, the five aforementioned upregulated CYP unigenes belonged to the CYP 4 and 6 families, and those two family genes have been implicated in insecticide resistance more often than any other P450 family [22,29]. These genes are unique to insects and play important roles in the metabolism and detoxification of pesticides [22], and it has been suggested that overexpression of unigenes in these two families was involved in insecticide tolerance and detoxification in *S. avenae*. In *B. odoriphaga*, CYP6FV12 showed different fold changes in different life stages when exposed to imidacloprid and was confirmed to be related to *B. odoriphaga* resistance to imidacloprid [30], while CYP6CM1vQ was confirmed to be associated with a high level of imidacloprid resistance in *Bemisia tabaci* [31]. Four other upregulated unigenes were only differentially expressed following treatment with imidacloprid for 3 h, and all of four of these were annotated as the CYP6A subfamily gene, which indicated that this subfamily gene may be important to *S. avenae* detoxification to imidacloprid. Therefore, although our results demonstrated that overexpression of CYP 4 and 6 family genes associated with the detoxification of imidacloprid and chlorpyrifos in *S. avenae*, whether these P450s can metabolize imidacloprid and chlorpyrifos needs further research.

ABC transporters play an important role in the detoxification process phase III, which can transport the polar compounds or conjugates out of the cell [32]. ABC transporters have been associated with imidacloprid and chlorpyrifos resistance in insects [33,34]. Eight ABCB/C/D/G subfamily transporter genes in imidacloprid and chlorpyrifos resistant strains of *Laodelphax striatellus* were significant upregulated compared with a susceptible strain [35], these results suggest that ABC transporters might be involved in resistance to multiple insecticides in *L. striatellus.* Two out the five ABC transporter genes analyzed in *Anopheles gambiae* were downregulated after the 48h exposure of permethrin [36]. In our study, six of the nine up-regulated ABC transporters belonged to C and G subfamilies. ABC transporter possessed a larger group of DEUs induced by imidacloprid and chlorpyrifos than the groups of P450, GST, and carboxylesterase, and more than two of three ABC transporter unigenes are downregulated after pesticides treatments. Thus, ABC transporter may play an important role in the detoxification process and insecticide tolerance of *S. avenae*.

In addition to insecticide detoxification, target site sensitivity and decreased penetration are important to insect tolerance to pesticides. In this study, no DEUs related to target site sensitivity were found, but dozens of penetration related DEUs were identified. Increased insecticides cuticular penetration, including cuticle thickening and alteration of cuticle composition, have previously been described [37]. In *Culex pipiens pallens*, cuticle protein played an important role in deltamethrin resistance [38], and CPLCG5 encoded a cuticle protein that participated in pyrethroid resistance by inducing rigidity and increasing the thickness of the cuticle [39]. There were five cuticle protein genes differentially expressed in deltamethrin-resistant *C. pipiens pallens* when compared with susceptible strains, with cuticle protein CP14.6 precursors found to be overexpressed in the deltamethrin-resistant strain. This may support the hypothesis that mosquitoes can protect themselves from insecticides by regulating cuticles, which finally leads to cuticular resistance [40]. In the present study, after exposure to insecticides, the largest group of DEUs was cuticle protein-related unigenes, and most of them were down regulated. This is also happening in *C. pipiens pallens*. In the 30 differentially expressed proteins identified by deltamethrin-resistant strain compared with the deltamethrin-susceptible strain, five out of 30 proteins are cuticle-related protein, and four out of five are downregulated [40]. Mevinphos resistance strains comparing with susceptible strains in *P. xylostella*, 12 out of 16 differentially expressed cuticle protein transcripts are downregulated [41]. Therefore, our study indicates that cuticle proteins may play an important role in metabolism or tolerance to insecticides by *S. avenae*, but how cuticle proteins are involved in the process of cuticle alterations, its alterations of cuticle structure or composition, and how to slow down the penetration of insecticides requires further research.

Trypsin-related genes accounted for a large group of DEUs in *B. oriphaga* under insecticide stress [6] and were found to be highly expressed in *C. pipiens pallens* deltamethrin-resistant strains [42]. Following exposure to triazophos, imidacloprid, chlorpyrifos, and abamectin, trypsin expression was upregulated in *Sodatella furcifera* [43]. In the present study, trypsin-related genes accounted for the largest group of upregulated unigenes under insecticide stress for 36 h, indicating that trypsin may be related to the response to stress induced by insecticides in *S. avenae*. In spirotetranmat- and thiamethoxam-resistant strains of *A. gossypii*, UGT was significantly upregulated relative to the susceptible strains [44,45]. In the present study, most of the UGTs were downregulated, while only one UGT was upregulated after exposure to insecticides, and all of the DEUs of UGT were assigned to KEGG as drug metabolism genes. In a female *Spodoptera littoralis*, exposure to a pheromone or plant odorant led to differential downregulation of the transcription levels of two UGTs specifically expressed in antennae [46]. Because UGTs played an important role in a variety of physiological and biochemical processes in insects, including detoxification of substrates (such as plant allelochemicals and insecticides) [47,48], our results indicated that UGTs may play a role in the tolerance and detoxification of insecticides; however, further study is needed to confirm these findings.

Sequencing by treatment with an insecticide to observe up- or downregulation of certain enzyme or receptor genes is the first step in understanding whether they are involved in pest resistance. Based on the analysis of unigenes that showed significant differences in the expression in response to these pesticides, it is concluded that the types and numbers of DEUs increased with the increased treatment time, while the differential unigene expression in response to different agents at the same time did not vary greatly. These findings may indicate that the production of *S. avenae* tolerance of insecticides does not occur via regulation by a single gene, but rather a result of joint regulation by multiple genes.

## 5. Conclusions

In this study, the adult transcriptome of *S. avenae* was sequenced, after which the unigenes database was assembled and this is the first time the annotation to different databases in *S. avenae* has occurred. The unigenes involved in responding to two insecticides, chlorpyrifos and imidacloprid, after different exposure times, were then identified and analyzed. The transcriptome assembly results provide a substantial contribution to the existing sequence resources for *S. avenae*. The analysis of DEUs responding to insecticides could provide a substantial foundation for research regarding the tolerance and detoxification mechanisms of *S. avenae*. The upregulated expression of cytochrome P450 genes may be important to pesticide detoxification in *S. avenae.* However, further investigation of the DEUs related to insecticide tolerance and detoxification is needed to determine if they can be used as molecular targets to explore novel approaches to control *S. avenae*.

## Figures and Tables

**Table 1:** Bioassay results of chlorpyrifos and imidacloprid to *Sitobion avenae.*

Insecticide	Regression Equation	LC_50_ (mg/L)	95% Confidence Interval	LC_10_ (mg/L)	95% Confidence Interval	X^2^	*p*-Value
chlorpyrifos	y = −0.89+1.39x	3.93	2.76–5.13	0.47	0.17–0.88	3.90	0.27
imidacloprid	y = −3.23+1.23x	429.90	172.80–784.54	38.90	1.43–115.15	10.16	0.038

Y: probit value of mortality minus 5; X: transformed pesticide concentration using the base 10 logarithm.

**Table 2:** Most significant differentially expressed unigenes (DEUs) following treatment with imidacloprid for 36 h versus control.

Unigene ID	Fold Change	Annotation
DN58420_c0_g1	4.96	anthranilate synthase component 1
DN68934_c1_g1	4.61	no result
DN56676_c0_g1	4.60	ribosomal large subunit pseudouridine synthase C
DN69579_c4_g1	4.53	WD repeat-containing protein 6 isoform X2
DN63141_c10_g2	3.95	uncharacterized family 31 glucosidase KIAA1161
DN64362_c2_g2	3.95	uncharacterized protein LOC111041562
DN59858_c0_g2	3.77	putative signal recognition particle protein
DN58147_c0_g1	3.64	regucalcin like isoform X1
DN69340_c3_g2	3.57	no result
DN62276_c7_g3	3.52	uncharacterized protein LOC111032214
DN62849_c0_g1	−4.39	uncharacterized protein LOC100169299
DN63539_c2_g2	−3.99	uncharacterized protein LOC111038926
DN59661_c1_g1	−3.98	gamma glutamyl transpeptidase 1 isoform X2
DN66753_c0_g1	−3.96	gamma glutamyl transpeptidase 1 isoform X2
DN61744_c1_g4	−3.78	uncharacterized protein LOC100574123
DN62043_c5_g2	−3.73	calphotin like
DN61971_c3_g5	−3.58	calphotin like
DN60187_c0_g1	−3.50	RNA binding protein 14
DN62043_c4_g1	−3.44	MAGE like protein 2
DN67869_c1_g3	−3.43	phytoene desaturase

**Table 3:** Most significant DEUs following treatment with chlorpyrifos for 36 h versus control.

Unigene ID	Fold Change	Annotation
DN58420_c0_g1	4.41	trpE
DN56676_c0_g1	4.11	ribosomal large subunit pseudouridine synthase C like
DN63141_c10_g2	4.07	uncharacterized family 31 glucosidase KIAA1161 like
DN69579_c4_g1	3.59	WD repeat-containing protein 6 isoform X2
DN58900_c5_g1	3.53	no result
DN68934_c1_g1	3.47	no result
DN58147_c0_g1	3.26	regucalcin like isoform X1
DN59687_c3_g4	3.17	uncharacterized protein LOC108376199
DN69016_c3_g2	3.05	no result
DN59858_c0_g	2.97	putative signal recognition particle protein
DN61971_c3_g5	−4.04	calphotin like
DN63539_c2_g2	−3.98	uncharacterized protein LOC111038926
DN62043_c5_g2	−3.94	calphotin like
DN66074_c0_g1	−3.80	MAGE-like protein 2
DN62043_c4_g1	−3.71	MAGE-like protein 2
DN61744_c1_g4	−3.49	uncharacterized protein LOC100574123
DN60266_c0_g1	−3.43	probable NADP-dependent mannitol dehydrogenase
DN66753_c0_g1	−3.35	UGT 2C1 like
DN61958_c0_g1	−3.33	protein-glutamate *O*-methyltransferase like
DN59661_c1_g1	−3.30	gamma-glutamyl transpeptidase 1 isoform X2

**Table 4:** Insecticide tolerance-related unigenes.

Gene Type	Unigenes Number in De Novo Database	IM 15 min vs. CK		IM 3 h vs. CK		IM 36 h vs. CK		CH 15 min vs. CK		CH 3 h vs. CK		CH 36 h vs. CK	
		Up	Down	Up	Down	Up	Down	Up	Down	Up	Down	Up	Down
Glutathione s-transferase	90	0	0	0	0	1	1	0	0	0	0	0	1
Carboxylesterase	81	0	0	0	0	0	6	0	0	0	0	0	7
Cytochrome P450	198	1	1	7	0	5	6	1	1	1	1	5	6
NADH dehydrogenase	135	0	0	0	0	1	0	1	0	1	0	1	1
Trypsin	218	0	0	2	3	15	17	1	0	1	0	8	12
Superoxide dismutase	26	0	0	0	0	1	0	0	0	0	0	0	2
ABC transporter	381	0	0	0	1	8	21	0	0	0	1	8	17
Cuticle protein	120	0	5	0	0	4	46	0	5	0	4	3	40
UGT	112	0	0	0	3	1	23	0	3	0	6	0	18
Acetylcholine receptor	12	0	0	0	1	2	1	0	0	0	0	0	0
Chloride channel	11	0	0	0	0	1	0	0	0	0	0	0	0

CK: control; CH: chlorpyrifos; IM: imidacloprid; and 15 min, 3 h, and 36 h: treatment with chlorpyrifos or imidacloprid for 15 min, 3 h, and 36 h, respectively.

## Supplementary Materials

The following are available online at https://www.mdpi.com/2073-4425/10/12/951/s1, File S1: R code for detect differential expression of unigenes; File S2: The primers to confirm unigenes and qRT-PCR; File S3: The amplification efficiencies of primers for qRT-PCR; File S4: Dose-response curves of *S. avenae* to chlorpyrifos and imidacloprid; File S5: Statistics of clean reads after RNA-Seq; File S6: Size distribution of unigenes; File S7: KEGG annotation and pathways of the *S. avenae* transcriptome; File S8: Cluster of orthologous groups functional classification of the *S. avenae* transcriptome; File S9: DEUs up- or downregulated in response to different treatments; File S10: GO enrichment analysis of DEUs; File S11: KEGG enrichment analysis of DEUs; File S12: Insecticide tolerance-related unigenes.
